# Tinned Fruit Consumption and Mortality in Three Prospective Cohorts

**DOI:** 10.1371/journal.pone.0117796

**Published:** 2015-02-25

**Authors:** Erlend T. Aasheim, Stephen J. Sharp, Paul N. Appleby, Martin J. Shipley, Marleen A. H. Lentjes, Kay-Tee Khaw, Eric Brunner, Tim J. Key, Nicholas J. Wareham

**Affiliations:** 1 Medical Research Council Epidemiology Unit, University of Cambridge, Cambridge, United Kingdom; 2 Cancer Epidemiology Unit, Nuffield Department of Population Health, University of Oxford, Oxford, United Kingdom; 3 University College London, Department of Epidemiology and Public Health, London, United Kingdom; 4 Department of Public Health and Primary Care, University of Cambridge, Cambridge, United Kingdom; Cardiff University, UNITED KINGDOM

## Abstract

Dietary recommendations to promote health include fresh, frozen and tinned fruit, but few studies have examined the health benefits of tinned fruit. We therefore studied the association between tinned fruit consumption and mortality. We followed up participants from three prospective cohorts in the United Kingdom: 22,421 participants from the European Prospective Investigation into Cancer and Nutrition (EPIC)-Norfolk cohort (1993–2012), 52,625 participants from the EPIC-Oxford cohort (1993–2012), and 7440 participants from the Whitehall II cohort (1991–2012), all reporting no history of heart attack, stroke, or cancer when entering these studies. We estimated the association between frequency of tinned fruit consumption and all cause mortality (primary outcome measure) using Cox regression models within each cohort, and pooled hazard ratios across cohorts using random-effects meta-analysis. Tinned fruit consumption was assessed with validated food frequency questionnaires including specific questions about tinned fruit. During 1,305,330 person years of follow-up, 8857 deaths occurred. After adjustment for lifestyle factors and risk markers the pooled hazard ratios (95% confidence interval) of all cause mortality compared with the reference group of tinned fruit consumption less often than one serving per month were: 1.05 (0.99, 1.12) for one to three servings per month, 1.10 (1.03, 1.18) for one serving per week, and 1.13 (1.04, 1.23) for two or more servings per week. Analysis of cause-specific mortality showed that tinned fruit consumption was associated with mortality from cardiovascular causes and from non-cardiovascular, non-cancer causes. In a pooled analysis of three prospective cohorts from the United Kingdom self-reported tinned fruit consumption in the 1990s was weakly but positively associated with mortality during long-term follow-up. These findings raise questions about the evidence underlying dietary recommendations to promote tinned fruit consumption as part of a healthy diet.

## Introduction

Dietary guidelines recommend eating 5 or more portions of fruit and vegetables daily to promote healthy eating and reduce the burden from cardiovascular disease, cancer and other chronic conditions in the general population [1] [2]. These recommendations are the result of translation of research evidence into pragmatic advice to improve public health. A limitation of this research is the difficulty in measuring dietary exposures accurately. In the case of fruit consumption, observational studies often do not describe whether the dietary assessment method distinguished between different fruit products, such as between fresh whole fruits and tinned fruit [3] [4] [5] [6] [7] [8] [9]. Dietary advice to increase fruit consumption nevertheless includes fresh, frozen, dried and tinned fruit as options in a healthy diet [1] [2].

Tinned fruit often contains added sugar [1] [2] and may have reduced concentrations of heat-liable nutrients following the canning procedure [10]. Historically canned foods sometimes caused acute lead intoxication through migration of lead from cans into food [11]. Today canned foods are recognized as a major source of human exposure to bisphenol A [12], a chemical which is used to coat the insides of food cans to prevent corrosion and might influence health through its endocrine-disrupting properties. Urinary bisphenol A concentrations have been positively associated with coronary artery disease in the cross-sectional National Health and Nutrition Examination Survey (NHANES) [13] and prospectively in participants followed for 10.8 years on average in the European Prospective Investigation into Cancer and Nutrition (EPIC)-Norfolk cohort [14].

The evidence of health benefits from eating tinned fruit products has to our knowledge not previously been examined. To inform dietary guidelines we therefore investigated the relationship between tinned fruit consumption and mortality. We also examined the relationship with mortality from cardiovascular causes and from cancer, the key targets for dietary prevention. We analyzed data from three prospective United Kingdom (UK) based cohorts, each using identical, specific methods to measure tinned fruit consumption.

## Methods

### Study population

We used data from three prospective cohorts, EPIC-Norfolk, EPIC-Oxford, and Whitehall II. The cohorts have been described in detail elsewhere [15] [16] [17]. EPIC-Norfolk enrolled 25,639 people aged 39–79 y among residents in Norfolk during 1993–1997 [15]. EPIC-Oxford enrolled 65,411 people aged 20–97 y among the general population (recruited from general practice) and health-conscious individuals, including a high proportion of vegetarians, throughout the UK (recruited by post) during 1993–2001 [16]. Whitehall II originally enrolled 10,308 London-based office staff aged 30–55 y during 1985–1988 [17], and a detailed dietary assessment was completed at the phase 3 visit in 1991–1993 [18] [19].

We considered participants as eligible for our study if they had completed a valid food frequency questionnaire (FFQ) with known tinned fruit consumption, had valid follow-up data (EPIC-Norfolk, N = 24,781; Whitehall II, N = 8360) and (for EPIC-Oxford, N = 54,133) were aged <90 years. We excluded people who reported a baseline history of heart attack, stroke, or cancer (N = 2360 from EPIC-Norfolk, 1508 from EPIC-Oxford, and 201 from Whitehall II). After exclusions for missing data, the resulting study sample available for analysis consisted of 22,421 participants from EPIC-Norfolk, 52,625 from EPIC-Oxford, and 7440 from Whitehall II.

### Assessment of dietary intake

Data on tinned fruit consumption were collected at enrolment for EPIC-Norfolk and EPIC-Oxford, and at the phase 3 visit for Whitehall II. Dietary intake was assessed using semi-quantitative FFQs, which instructed participants to specify how often on average they had eaten 130 different food items (Whitehall II: 127 items) over the past year [18] [20] [21]. Each FFQ included a fruit category with 11 items: apples, pears, oranges, grapefruit, bananas, grapes, melons, peaches, strawberries, tinned fruit, and dried fruit (S1 Fig.) [22]. Participants could choose from nine frequency alternatives ranging from “Never or less than once/month” up to “6+ per day”. To estimate grams of food consumed per day, the frequency of consumption was multiplied by a standard serving weight for each food. Energy (kJ/day) and alcohol (g/day) intakes were calculated using McCance & Widdowson's food composition tables. The FFQs were developed based on the FFQ used in the Nurses' Health Study, and were adapted to British diets using information from the National Food Survey [18] [22]. The FFQs have been extensively validated [18] [20] [22] [23].

### Non-dietary covariates

Participants completed health and lifestyle questionnaires developed for each cohort. The questionnaires included questions about the participants' medical history, medication use, family history, smoking status, alcohol intake, education level, occupation, and physical activity level, as described previously [15] [16] [17]. The physical activity index developed for EPIC-Norfolk was validated in individually calibrated heart rate monitoring studies [24]. Body mass index (BMI) was calculated as weight divided by height squared (kg/m^2^). Height and weight were measured by study personnel in EPIC-Norfolk [15] and Whitehall II [17], but were generally based on self-report in EPIC-Oxford [16]. A validation study conducted in 4808 participants from EPIC-Oxford showed high Spearman rank correlations between self-reported and measured height and weight (*r*_s_ > 0.9) [25].

### Outcome

The vital status of participants was ascertained by linkage with the UK National Health Service Central Register. Death certificates were coded by nosologists according to the International Classification of Disease (ICD). Cardiovascular death was defined with ICD 401–448 (ICD9) or ICD I10–I79 (ICD 10) as underlying cause of death, which includes coronary heart disease, stroke and other vascular causes. Cancer death, excluding non-melanoma skin cancer was defined with ICD-9 140–208 (excluding 173) or ICD-10 C00–C97 (excluding C44) as underlying cause of death. Other deaths were classified as non-cardiovascular, non-cancer deaths. In Whitehall II the cause of death was unknown for five participants, who were excluded from analysis of cause specific mortality.

It can be assumed that ascertainment of all cause mortality through death registers is accurate. In a validation study of coronary artery disease cases ascertained from death certificates in EPIC-Norfolk participants, confirmation of the cause of death was sought from general practices, hospital notes, and post-mortem reports; the diagnosis (based on WHO criteria [26]) was confirmed in 38 of 39 cases [27]. Death certificates and hospital record linkage have also been shown to have a high accuracy for identifying stroke cases in EPIC-Norfolk participants [28]. These findings indicate that death certificates can provide a valid method for identifying cardiovascular deaths in the populations studied.

### Ethics

Ethical permission for the EPIC-Norfolk study was obtained from The Norfolk and Norwich Hospital Ethics Committee, for the EPIC-Oxford study from the multicentre research ethics committee (MREC/02/0/90), and for The Whitehall II study from the University College London ethics committee. All participants in these cohorts provided written informed consent to be included.

### Statistics

We calculated participants' person-years of follow-up from the date of assessment of tinned fruit consumption to the date of death, or end of follow-up (31.12.2012 for EPIC-Norfolk, 30.09.2012 for EPIC-Oxford, and 30.08.2012 for Whitehall II), whichever came first. We conducted analyses separately for each cohort using Cox regression models to estimate hazard ratios and 95% CIs for all cause and cause specific mortality comparing four frequency categories of tinned fruit intake: never or less than 1 serving per month (reference category), 1 to 3 servings per month, 1 serving per week, and 2 or more servings per week (coded as 0, 1, 2 and 3, respectively). Assuming a dose-dependent effect of tinned fruit consumption on mortality, we also calculated hazard ratios and P values for linear trend relating to a one serving per week increase in tinned fruit consumption. We fitted two main models, one adjusting for sex and age (as underlying time scale) and a multivariable model additionally adjusting for lifestyle and risk factors measured at baseline, including BMI, smoking status, alcohol intake, physical activity level, socioeconomic status, prior history of diabetes, use of anti-hypertensive or lipid-lowering drugs, family history of disease, and energy intake. Covariates included in the multivariable model differed slightly between cohorts because of differences in the health and lifestyle questionnaires used. Full information on the covariates used for each cohort is given in footnotes to the table showing results for the primary outcome.

We used random-effects meta-analysis to estimate pooled hazard ratios across cohorts, and calculated *I* ^2^ values to estimate the extent of heterogeneity in estimated hazard ratios between cohorts [29]. To assess potential effect modification we introduced multiplicative interaction terms for sex, age at baseline (<60 or ≥60 y), and BMI (non-obese: <30 kg/m^2^ or obese: ≥30 kg/m^2^) with the tinned fruit variable (in four frequency categories) into the multivariable model for all cause mortality and tested the null hypothesis that the interaction parameters were zero (on a log scale) using a likelihood ratio test.

To assess potential reverse causality we conducted analyses excluding participants who died in the first 2 y of follow-up. We also conducted analyses excluding participants with unusually high (>40 kg/m^2^) or low (<18.5 kg/m^2^) BMIs under the hypothesis that they might follow unusual diets or were malnourished due to illness; and excluding participants with intermediate outcomes (diabetes mellitus, or drug-treated hypertension or hyperlipidemia) that can be hypothesized to be on the causal pathway between diet and cardiovascular disease. We further assessed the impact on the results of including additional covariates in the multivariable models, by adding plasma vitamin C concentration, social class, and ethnicity for EPIC-Norfolk; and by adding education level for Whitehall II, where employment grade was used as a marker of socioeconomic status in the primary analyses. Because some participants in EPIC-Norfolk and Whitehall II had completed new FFQs at follow-up visits (EPIC-Norfolk: 10,973 participants during 1998–2003; and Whitehall II: 4784 during 1997–1999 and 4959 during 2002–2004), we also conducted additional analyses where we updated data on tinned fruit consumption over time.

In a dietary replacement analyses we estimated the effect on all cause mortality of replacing 1 serving of non-tinned fruit with 1 serving of tinned fruit by introducing individual variables for each of the 11 fruit types assessed by the FFQ (coded in number of servings per week) and a total fruit variable (created as the sum of the individual fruit variables) into the multivariable model for all cause mortality; we then removed an individual fruit variable (e.g. apples), and in that model we interpreted the hazard ratio for tinned fruit as the effect of replacing 1 serving of the removed fruit variable (e.g. apples) with 1 serving of tinned fruit. This analysis assumes that the total number of fruit servings a person consumes is constant, and addresses the practical question of replacing 1 serving of tinned fruit with another fruit (which is displaced from the diet) while simultaneously adjusting for the consumption of other fruits and the other covariates in the multivariable model [30].

We performed the analyses in Stata 12.1 (StataCorp LP, College Station, TX, USA) and SAS 9.2 (SAS Institute Inc., Cary, NC, USA).

## Results

### Baseline characteristics

Baseline characteristics of participants in EPIC-Norfolk, EPIC-Oxford and Whitehall II are shown pooled in Table 1 and by cohort in S1 Table. The majority of participants (61.5%) consumed less than one serving of tinned fruit per month. Compared to participants reporting less frequent tinned fruit consumption, participants reporting more frequent tinned fruit consumption tended to be male, older, have higher BMIs, be physically inactive, have lower educational qualifications, and have higher energy intakes. Frequent tinned fruit consumers were also less likely to be current smokers and had lower alcohol intakes.

**Table 1:** Pooled participant characteristics at baseline according to tinned fruit consumption.^a^

**Characteristic**	**Frequency of tinned fruit consumption^b^**			
	<1 per month N = 50,727	1–3 per month N = 19,868	1 per week N = 8147	≥2 per week N = 3744
Age (y)	47.3 ± 12.1	49.8 ± 12.3	51.3 ± 12.0	51.9 ± 12.8
Male, %	30.6	35.9	38.6	38.9
Current smoker, %	12.5	10.6	10.5	9.6
Education, O level or less^c^, %	34.4	39.9	46.2	48.1
Alcohol consumption (grams/day)	10.1 ± 12.8	7.8 ± 11.0	6.8 ± 9.9	6.2 ± 9.6
Total energy intake (MJ/day)	7.9 ± 2.2	8.7 ± 2.3	9.1 ± 2.5	9.4 ± 2.5
Body mass index (kg/m^2^)	24.2 ± 3.8	24.8 ± 3.8	25.1 ± 3.9	25.2 ± 4.0
Physically inactive, %	23.9	24.9	26.5	29.4
Prior diabetes mellitus, %	1.3	1.5	2.0	2.7

Values are means ± SDs or percentages.

^a^ Participant characteristics by cohort are shown in S1 Table.

^b^ Consumption of tinned fruit relates to one medium serving (defined as 120 g).

^c^ In the United Kingdom, the Ordinary (O) level qualification was normally reached at 16 years of age; this qualification was replaced by the General Certificate of Secondary Education (GCSE) in 1988.

8857 deaths occurred during 1,305,330 person years of follow-up: EPIC-Norfolk, 4759 deaths during 353,887 person years (median 17 years follow-up); EPIC-Oxford, 3399 deaths during 819,644 person years (median 16 years follow-up); and Whitehall II, 698 deaths during 149,630 person years (median 20 years follow-up). EPIC-Norfolk participants, who were older than participants in the other cohorts, had higher death rates during follow-up (S2 Table). Cancer was the most common cause of death in the cohorts.

### All cause mortality

More frequent tinned fruit consumption was weakly associated with increased mortality in both age and sex adjusted analyses and in multivariable analyses (Table 2). In pooled multivariable adjusted analyses the hazard ratios (95% CI) of all cause mortality compared with the reference category of consuming less than one serving of tinned fruit per month were: 1.05 (0.99, 1.12) for one to three servings per month, 1.10 (1.03, 1.18) for one serving per week, and 1.13 (1.04, 1.23) for two or more servings per week; no important heterogeneity in estimated hazard ratios between cohorts was identified (I^2^ ranged from 0% to 28.6%). The hazard ratio for mortality for a one serving per week increase in tinned fruit consumption was 1.03 (1.01, 1.04) (*p* = 0.003), *I* ^2^ = 0.2%. There were no significant interactions between tinned fruit consumption and sex, age, or BMI in relation to all cause mortality in any of the cohorts.

**Table 2:** Hazard ratios (95% CIs) for all cause mortality by tinned fruit consumption.

	**Frequency of tinned fruit consumption**			One serving per week increase		
	<1/month	1–3/month	1/week	≥2/week	Linear trend	p-Value
**EPIC-Norfolk, 1993–2012**						
Deaths/participants, N (%)	2134/11655 (18.3)	1423/6260 (22.3)	812/3152 (25.8)	373/1355 (28.9)		
Age and sex adjusted hazard ratio	1.00	1.05 (0.99, 1.13)	1.15 (1.06, 1.24)	1.19 (1.07, 1.32)	1.04 (1.02, 1.06)	
Multivariable adjusted hazard ratio^a^	1.00	1.02 (0.95, 1.09)	1.13 (1.04, 1.23)	1.16 (1.04, 1.30)	1.03 (1.01, 1.06)	
**EPIC-Oxford, 1993–2012**						
Deaths/participants, N (%)	1982/34795 (5.7)	869/11594 (7.5)	341/4195 (8.1)	207/2041 (10.1)		
Age and sex adjusted hazard ratio	1.00	1.11 (1.02, 1.20)	1.12 (1.00, 1.26)	1.19 (1.03, 1.38)	1.03 (1.00, 1.06)	
Multivariable adjusted hazard ratio^a^	1.00	1.11 (1.02, 1.21)	1.07 (0.95, 1.21)	1.09 (0.95, 1.27)	1.01 (0.98, 1.05)	
**Whitehall II, 1991–2012**						
Deaths/participants, N (%)	397/4277 (9.3)	189/2015 (9.4)	77/800 (9.6)	35/348 (10.1)		
Age and sex adjusted hazard ratio	1.00	0.94 (0.79, 1.12)	0.97 (0.76, 1.24)	1.01 (0.71, 1.42)	0.98 (0.90, 1.07)	
Multivariable adjusted hazard ratio^a^	1.00	1.00 (0.83, 1.19)	0.98 (0.76, 1.27)	1.04 (0.73, 1.48)	0.99 (0.91, 1.08)	
**Pooled results**						
Age and sex adjusted hazard ratio^b^	1.00	1.06 (0.99, 1.13)	1.13 (1.06, 1.20)	1.18 (1.08, 1.28)	1.03 (1.02, 1.05)	<0.001
Multivariable adjusted hazard ratio^b^ ^,^ ^c^	1.00	1.05 (0.99, 1.12)	1.10 (1.03, 1.18)	1.13 (1.04, 1.23)	1.03 (1.01, 1.04)	0.003

EPIC, European Prospective Investigation into Cancer and Nutrition.

^a^ All multivariable models adjusted for the following factors at baseline: sex, age (as underlying time variable), alcohol intake (four categories), physical activity level (four categories from low to high), prior diabetes (yes or no), smoking status (never, former or current in EPIC-Norfolk and Whitehall II; for EPIC-Oxford current smoking was divided into light or heavy smoker with the latter defined as ≥15 cigarettes smoked per day), body mass index (continuous for EPIC-Norfolk and Whitehall II and divided into five categories for EPIC-Oxford: <20.0, 20.0–22.4, 22.5–24.9, 25.0–27.4, and ≥27.5 kg/m^2^), socio-economic status (education level in four categories for EPIC-Norfolk and EPIC-Oxford, and employment grade in three categories for Whitehall II), energy intake (total energy intake for EPIC-Norfolk and EPIC-Oxford, and ratio of reported energy intake to estimated energy expenditure for Whitehall II). In addition, EPIC-Norfolk adjusted for antihypertensive drug use (yes or no), lipid lowering drug use (yes or no), family history of heart attack (yes or no), and family history of cancer (yes or no); EPIC-Oxford adjusted for long-term medical treatment (yes or no), parental history of heart attack or cancer (yes or no), and stratified hazard ratios by method of recruitment (general practice or post); and Whitehall II adjusted for antihypertensive drug use (yes or no), lipid lowering drug use (yes or no), ethnicity (white, south asian, black, other), and diet pattern (healthy, sweet, Mediterranean-like, unhealthy).

^b^ Pooled results were obtained in a random-effects meta-analysis of the log of the adjusted hazard ratios from individual cohorts.

^c^ Between-study heterogeneity measured by *I* ^2^ for the pooled multivariable adjusted hazard ratios were: 28.6% for one to three servings per month, 0% for one serving per week, 0% for two or more servings per week, and 0.2% for one serving per week increase.

### Cause specific mortality

**Fig 1:**
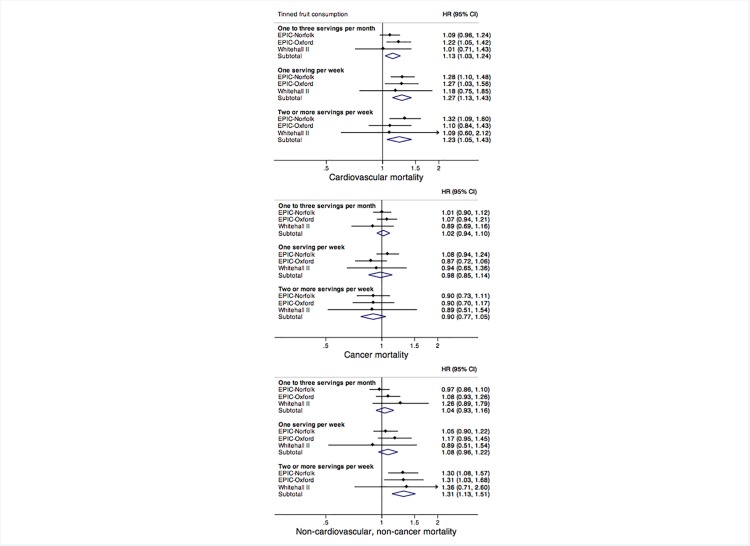
Hazard ratios (95% CIs) for cause-specific mortality associated with consumption of tinned fruit, compared to the reference category of less than one serving of tinned fruit per month. Pooled results were calculated in random-effects meta-analysis of the log of the hazard ratios from individual cohorts, which were obtained in multivariable models including the same covariates as for analysis of all cause mortality (see Table 2). Between-study heterogeneity measured by *I* ^2^ was, for cardiovascular mortality: 0% for one to three servings per month, 0% for one serving per week, and 0% for two or more servings per week; for cancer mortality 0% for one to three servings per month, 33.0% for one serving per week, and 0% for two or more servings per week; and for non-cardiovascular, non-cancer mortality: 21.2% for one to three servings per month, 0% for one serving per week, and 0% for two or more servings per week.

Analyses of cause specific mortality showed that tinned fruit consumption was associated with mortality from cardiovascular causes and from non-cardiovascular, non-cancer causes, but not from cancer (Fig. 1). For cardiovascular mortality the pooled multivariable adjusted hazard ratios compared with the reference category of consuming less than one serving of tinned fruit per month were: 1.13 (1.03, 1.24) for one to three servings per month, 1.27 (1.13, 1.43) for one serving per week, and 1.23 (1.05, 1.43) for two or more servings per week. For non-cardiovascular, non-cancer mortality the adjusted hazard ratios compared with consuming less than one serving of tinned fruit per month were: 1.04 (0.93, 1.16) for one to three servings per month, 1.08 (0.96, 1.22) for one serving per week, and 1.31 (1.13, 1.51) for two or more servings per week. There was no important heterogeneity in hazard ratios between cohorts.

### Sensitivity analyses

The association between tinned fruit consumption and all cause mortality was unaffected when we excluded participants with unusually high or low BMIs, participants reporting a history of diabetes, antihypertensive drug use, or lipid lowering drug use at baseline, and participants who died in the first 2 y of follow-up (S3 Table). Moreover, the association between tinned fruit consumption and all cause mortality was not attenuated when we added any of social class, ethnicity or plasma vitamin C level to the multivariable model for EPIC-Norfolk or when we added education level to the multivariable model for Whitehall II. Subgroup analyses showed suggestions of stronger associations between tinned fruit consumption and all cause mortality in women, obese and people aged 60 y or above compared to men, non-obese and people aged <60 y, respectively (S3 Table). Updating dietary exposure data during follow-up had little effect on the association between tinned fruit consumption and all cause mortality in EPIC-Norfolk, and strengthened the association in Whitehall II, compared to the primary analyses (S3 Table).

### Dietary replacement of non-tinned fruit with tinned fruit

**Fig 2:**
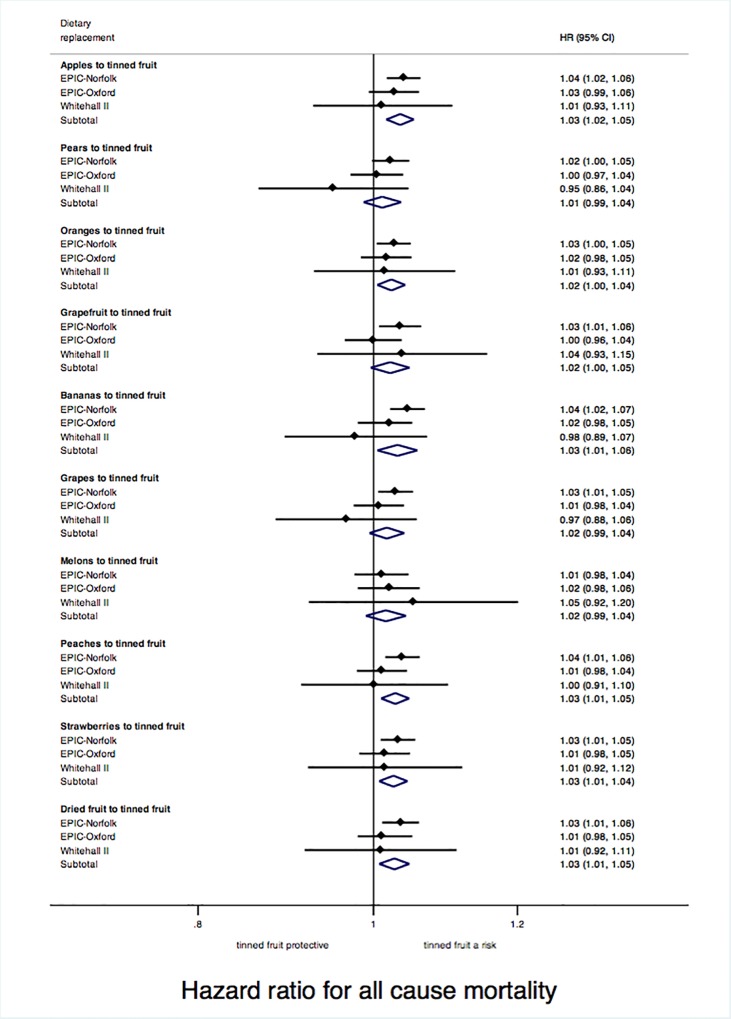
Hazard ratios (95% CIs) for all cause mortality associated with changing dietary intake from non-tinned fruits to tinned fruit. The effect on mortality of replacing 1 serving of tinned fruit with other types of fruit was estimated by introducing a variable for each fruit type assessed by the food frequency questionnaire (apples, pears, oranges, grapefruit, bananas, grapes, melons, peaches, strawberries, tinned fruit and dried fruit; each coded according to a participant´s intake in servings per week) and a total fruit variable (created as the sum of said fruit variables) into the multivariable model; an individual fruit variable (e.g. apples) was then removed, and in that model the hazard ratio for tinned fruit was interpreted as the effect of replacing 1 serving of the removed fruit variable (e.g. apples) with 1 serving of tinned fruit. Pooled results were obtained in a random-effects meta-analysis of the log of the multivariable adjusted hazard ratios from individual cohorts. Between-study heterogeneity measured by *I* ^2^ was: 0% for apples, 22.2% for pears, 0% for oranges, 14.7% for grapefruit, 29.6% for bananas, 20.7% for grapes, 0% for melons, 0% for peaches, 0% for strawberries, and 0% for dried fruit.

In dietary replacement analysis for the 10 non-tinned fruits, replacing consumption of the following fruits with tinned fruit was associated with significant increases in mortality: apples (hazard ratio 1.03; 95% CI 1.02, 1.05), oranges (1.02; 1.00, 1.04), bananas (1.03; 1.01, 1.06) peaches (1.03; 1.01, 1.05), strawberries (1.03; 1.01, 1.04) and dried fruit (1.03; 1.01, 1.05); there was no important heterogeneity across different cohorts (Fig. 2). Replacing pears, grapefruit, grapes, or melons with tinned fruit was associated with non-significant increases in mortality. The participants' consumption of these fruits is shown in S4 Table.

## Discussion

In this combined analysis of three prospective UK based cohorts we found no evidence of health benefits associated with tinned fruit consumption. Instead, participants who reported more frequent tinned fruit consumption at baseline had a moderately increased risk of mortality during follow-up, independently of lifestyle factors and other risk markers. Analysis of mortality by cause showed that this association was driven by cardiovascular deaths and non-cardiovascular, non-cancer deaths. Furthermore, in a dietary replacement analysis assuming that overall fruit consumption is constant, replacing consumption of several non-tinned fruits with tinned fruit was associated with moderate increases in all cause mortality.

### Comparison with previous studies

Few previous studies are available for direct comparison with the present study. Our study was possible because the FFQs in EPIC-Norfolk, EPIC-Oxford and Whitehall II asked specific questions about tinned fruit consumption. In some cohorts FFQs combine fresh, frozen and tinned fruits into single dietary exposures [31] [32], making it impossible to address research questions relating to tinned fruit consumption. Relationships between overall fruit intake and health outcomes have been investigated in many observational studies. Meta-analyses of cohort studies show that overall fruit consumption is associated with a small reduction in risk of coronary heart disease [33] [34]. In a recent study of 313,074 people followed for 8.4 y, where overall fruit consumption was associated with a minor reduction in ischemic heart disease mortality (relative risk 0.96 per 80 g/day increment), only fresh (not tinned) fruit was counted towards fruit consumption [35]. Several studies have found in subgroup analyses that different fruit products can have qualitatively different associations with clinical outcomes, and consumption of processed fruits has been associated with less favorable outcomes. In an analysis of three US cohorts, more frequent consumption of certain whole fruits (e.g. bananas) was associated with lower risk of incident type 2 diabetes, while frequent consumption of fruit juice products was associated with higher risk of incident type 2 diabetes [36]. Similarly, in 20,069 people followed for 10.3 y, consumption of raw fruits and vegetables showed a weak inverse association, while consumption of processed fruits and vegetables showed a weak positive association, with ischemic strokes [37]. Furthermore, in 65,226 people from nationally representative random samples of the noninstitutionalised population in England followed for a median of 7.7 years, consumption of frozen/canned fruit was associated with increased mortality (hazard ratio 1.17; 95% CI 1.07 to 1.28) per portion [38].

### Possible explanations for findings

There are several potential explanations for our findings. The association between tinned fruit consumption and mortality was stronger in EPIC-Norfolk than in the other cohorts, suggesting the association could have occurred by chance. Frequent consumers of tinned fruit tended to be male, older, report lower education level, have higher BMI, and more likely to have diabetes, and we did not adjust for factors such as income or dietary factors other than total energy intake; suggesting that residual confounding could potentially explain the identified association. Then again, frequent tinned fruit consumers were less likely to be current smokers, consistent with negative confounding. It has been hypothesized that fruit consumption in general could improve health outcomes through increased consumption of advantageous nutrients in fruit or through reduced consumption of disadvantageous nutrients in other foods, but these hypotheses have not been verified in randomized intervention trials [39] [40]. Tinned fruits often contain added sugar and consumption of added sugar may be associated with cardiovascular disease mortality [41]. Current dietary advice encourages people to buy fruit tinned in natural juice over tinned fruit containing added sugar [1] [2]. Canned foods can be contaminated with components of cans. In a randomized cross-over study, people consuming one serving of canned soup for 5 days showed a 1200% increase in urinary levels of bisphenol A compared to people consuming soup prepared without canned ingredients [42]. However, bisphenol A concentrations in tinned fruit (albeit higher than in fresh fruit) tend to be several-fold lower than in other canned foods [12]. Tinned fruits are the most acidic of canned foods [43] and could therefore more readily dissolve lead solder from food cans [44]. Food cans manufactured with lead solder appear to have been available to UK consumers up until the 1990s [45]. In the US Total Diet Study, lead concentrations in canned fruits were in the higher range compared to other foods during 1991–2005 [46]. In the Normative Aging Study, men in the highest tertile of bone lead content (a marker of cumulative lead exposure [47]) had increased risk of mortality from all causes (hazard ratio 2.52; 95% CI 1.17, 5.41) and particularly from ischemic heart disease (hazard ratio 8.37; 1.29, 54.4) compared with the lowest tertile of bone lead after adjustment for age, smoking and education [48]. It could be hypothesized that our findings reflect tinned fruit consumption during a time when lead solder was more widely used in the manufacture of food cans. Under such a hypothesis the association between tinned fruit and mortality might be strongest among older participants, consistent with our observations.

### Strengths and weaknesses

This study has limitations. We cannot infer that consumption of tinned fruit has adverse health consequences based on a single, observational investigation. Some inaccuracy in the assessment of tinned fruit consumption and classification of cause of death is inevitable [49]; but such inaccuracy is expected to be random and this would likely weaken any association between tinned fruit consumption and mortality. Because tinned fruit is recommended as a healthy food option it seems unlikely that participants would have underreported tinned fruit consumption due to perceived social desirability. The FFQs did not discriminate between different types of tinned fruit, and we could therefore not study specific tinned fruit products. In the FFQs the question about tinned fruit followed after questions for some other fruit categories, this could potentially lead to overestimation of consumption for these fruit categories since participants might have included tinned versions of these fruits when recording frequency of consumption. Although we did not identify significant statistical heterogeneity in pooled analyses for all cause mortality, statistical power to assess such heterogeneity was low. When considering the relevance of our data in other settings it should be noted that we examined UK based cohorts, which included predominantly white people who underwent a baseline examination in the 1990s. Since then, the effect of tinned fruit consumption on health could have changed if food cans are now manufactured differently.

Our study also has strengths. Because we analyzed data from prospective cohorts, any error in the measurement of tinned fruit consumption and covariates during follow-up is independent of mortality ascertainment. We used FFQs with specific questions in relation to tinned fruit consumption, which helps to minimize misclassification of this dietary exposure. Furthermore, we report long-term data from cohorts with highly accurate ascertainment methods for the primary outcome; we adjusted for major known confounders; and we performed several sensitivity analyses to test the robustness of our findings.

### Implications

If replicated our finding of an association between tinned fruit consumption and mortality will have several implications. If consumption of tinned fruit has different associations with health outcomes from consumption of non-tinned fruit then this might have influenced previous research combining tinned fruit and non-tinned fruit into a single dietary exposure; although any such influence may be expected be small, According to the UK National Food Survey, average household consumption of tinned fruit declined during 1975–2000 [50], suggesting tinned fruit consumption is less widespread than before. However, if tinned fruit is not beneficial to health then this may be particularly disadvantageous to vulnerable groups. Consumption habits are influenced by the cost and availability of food. In the UK National Food Survey tinned peaches, tinned pears and tinned pineapple were among the cheapest fruit products available, and people with low incomes consumed more canned food than people with high incomes [51]. Indeed, the UK 5 A DAY campaign states 'stock up on canned fruit and vegetables to save money' [1]. In New York, shops in predominantly black neighbourhoods had less fresh fruit and more canned fruit available than predominantly white neighbourhoods [52]. Furthermore, tinned fruit may be provided to preschool children [53] and other groups as part of efforts to meet recommended dietary intakes of fruit.

In conclusion, we found no evidence to suggest a benefit on mortality from consumption of tinned fruit. Our study and previous analyses together raise questions about the wisdom of current dietary recommendations promoting consumption of tinned fruit as part of a healthy diet.

## Supporting Information

S1 Fig Excerpts from food frequency questionnaires, showing questions for fruit consumption. (DOCX)

S1 Table Participant characteristics at baseline by cohort. (DOCX)

S2 Table Mortality during follow-up. (DOCX)

S3 Table Sensitivity and subgroup analyses: Hazard ratios (95% CIs) for all cause mortality by tinned fruit consumption. (DOCX)

S4 Table Fruit consumption at baseline, shown in grams per day. (DOCX)
