# Supplementary material for: Tinned Fruit Consumption and Mortality in Three Prospective Cohorts
Source: PLoS One. 2015 Feb 25;10(2):e0117796. doi: 10.1371/journal.pone.0117796 (PMC4340615; doi:10.1371/journal.pone.0117796)
Supplement: S1 Fig — (DOCX) [file pone.0117796.s001.docx]

**Figure S1. Excerpts from food frequency questionnaires, showing questions for fruit consumption.**

EPIC-Norfolk


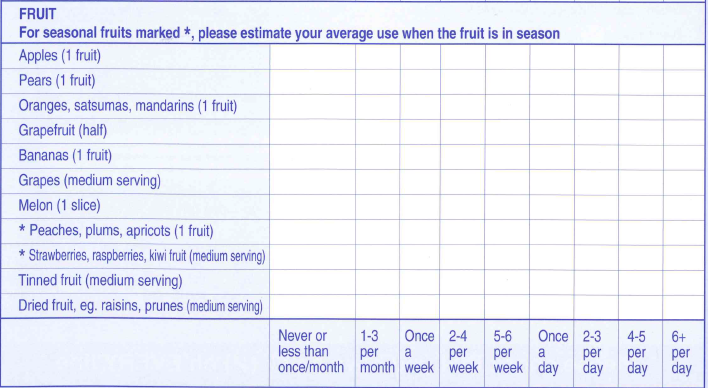


EPIC-Oxford


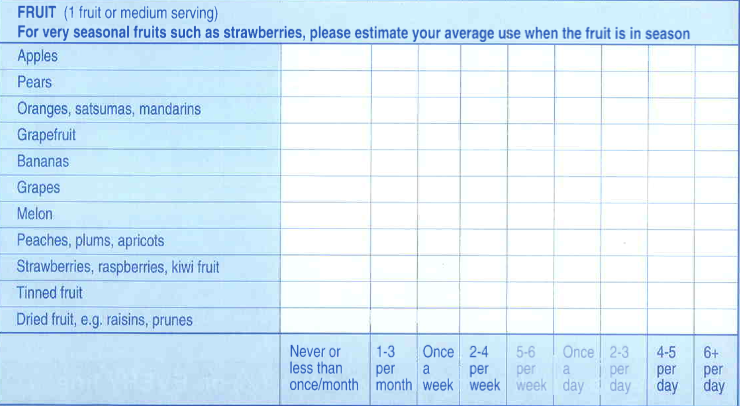


Whitehall II^1^


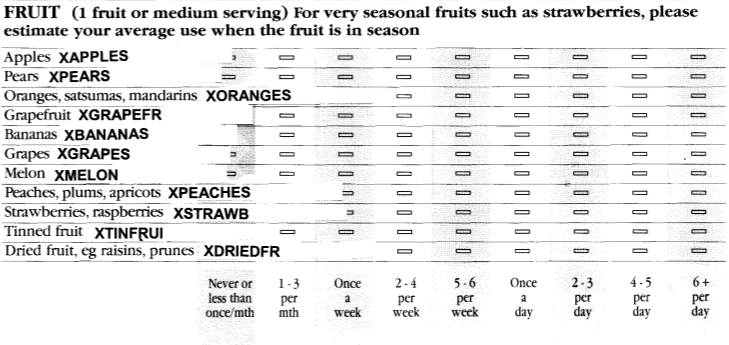


^1^ The labels 'XAPPLES', 'XPEARS' etc. were not included in the version given to participants.

**Table S1. Participant characteristics at baseline by cohort.**

| **Characteristic** | **Frequency of tinned fruit consumption** | | | |
| --- | --- | --- | --- | --- |
|  | <1 per month | 1-3 per month | 1 per week | ≥2 per week |
| **EPIC-Norfolk, 1993-1997** |  |  |  |  |
| Participants, N | 11,655 | 6259 | 3152 | 1355 |
| Male, % | 42.2 | 46.6 | 49.2 | 50.1 |
| Age (y) | 57.2 ± 9.1 | 58.9 ± 9.3 | 59.5 ± 9.2 | 60.9 ± 9.2 |
| Ethnicity, white, % | 99.7 | 99.6 | 99.7 | 99.4 |
| Body mass index (kg/m^2^) | 26.1 ± 3.9 | 26.4 ± 3.8 | 26.6 ± 3.9 | 26.6 ± 3.9 |
| Physically inactive, % | 27.6 | 29.9 | 30.7 | 36.2 |
| Current smoker, % | 12.5 | 11.3 | 10.9 | 9.0 |
| Education, none or primary, % | 31.4 | 38.3 | 44.6 | 46.9 |
| Prior diabetes mellitus, % | 1.8 | 2.0 | 2.3 | 3.4 |
| Antihypertensive drug use, % | 14.8 | 17.7 | 18.8 | 19.9 |
| Lipid lowering drug use, % | 1.0 | 1.0 | 1.3 | 1.5 |
| Parental history of heart attack, % | 36.3 | 36.2 | 35.5 | 35.5 |
| Parental history of cancer, % | 33.0 | 32.5 | 32.4 | 32.6 |
| Total energy intake (kJ/day) | 8110 ± 2331 | 8959 ± 2464 | 9417 ± 2683 | 9824 ± 2810 |
| Alcohol intake (g/day) | 8.3 ± 10.2 | 6.4 ± 8.8 | 5.4 ± 8.1 | 5.2 ± 7.6 |
| Plasma vitamin C (µmol/L) | 55 ± 20 | 52 ± 20 | 51 ± 20 | 52 ± 20 |
| **EPIC-Oxford, 1993-2001** |  |  |  |  |
| Participants, N | 34,795 | 11,594 | 4195 | 2041 |
| Male, % | 22.3 | 23.5 | 23.7 | 24.3 |
| Age (y) | 43.7 ± 13.4 | 44.9 ± 14.3 | 45.7 ± 14.6 | 46.4 ± 15.4 |
| Ethnicity, white, % | 97.8 | 98.2 | 98.4 | 98.6 |
| Body mass index (kg/m^2^) | 23.5 ± 3.7 | 23.9 ± 3.9 | 24.2 ± 4.0 | 24.2 ± 4.1 |
| Physically inactive, % | 23.3 | 24.1 | 25.8 | 27.1 |
| Current smoker, % | 12.2 | 9.9 | 9.9 | 9.7 |
| Education, O level or less, % | 11.6 | 15.3 | 20.5 | 20.7 |
| Prior diabetes mellitus, % | 1.2 | 1.3 | 2.0 | 2.6 |
| Receiving long-term medical treatment, % | 23.6 | 26.5 | 29.9 | 33.4 |
| Parental history of heart attack or cancer, % | 52.2 | 53.5 | 53.2 | 52.1 |
| Total energy intake (kJ/day) | 7778 ± 2158 | 8558 ± 2265 | 8857 ± 2336 | 9019 ± 2410 |
| Alcohol intake (g/day) | 10.5 ± 13.2 | 8.3 ± 11.5 | 7.3 ± 10.6 | 6.5 ± 10.4 |
| **Whitehall II, 1991-1993** |  |  |  |  |
| Participants, N | 4277 | 2015 | 800 | 348 |
| Male, % | 66.1 | 73.6 | 76.5 | 79.3 |
| Age (y) | 49.2 ± 6.0 | 49.7 ± 6.1 | 50.0 ± 6.0 | 49.6 ± 6.3 |
| Ethnicity, white, % | 90.6 | 91.3 | 95.0 | 96.0 |
| Body mass index (kg/m^2^) | 25.3 ± 3.8 | 25.2 ± 3.5 | 25.4 ± 3.6 | 25.1 ± 3.7 |
| Physically inactive, % | 18.5 | 14.3 | 15.1 | 16.7 |
| Current smoker, % | 15.0 | 12.2 | 12.4 | 11.8 |
| Education, none or primary, % | 35.6 | 39.6 | 41.2 | 43.4 |
| Low employment grade, % | 15.0 | 15.9 | 15.5 | 18.1 |
| Prior diabetes mellitus, % | 0.7 | 0.9 | 0.6 | 0.6 |
| Antihypertensive drug use, % | 6.2 | 5.8 | 7.8 | 6.3 |
| Lipid lowering drug use, % | 0.6 | 0.7 | 1.0 | 1.2 |
| Total energy intake (kJ/day) | 8152 ± 2314 | 9152 ± 2401 | 9540 ± 2434 | 9983 ± 2315 |
| Reported energy intake / estimated energy expenditure | 0.74 ± 0.22 | 0.82 ± 0.23 | 0.85 ± 0.25 | 0.89 ± 0.24 |
| Alcohol intake (g/day) | 13.0 ± 15.1 | 10.7 ± 14.2 | 9.3 ± 12.6 | 8.6 ± 11.5 |
| Alcohol intake: None, % | 16.4 | 19.8 | 22.9 | 24.7 |
| Alcohol intake: 1-8 g/day, % | 35.1 | 38.9 | 42.3 | 42.2 |
| Alcohol intake: 9-16(F)/24(M) g/day, % | 29.3 | 29.6 | 24.4 | 24.4 |
| Alcohol intake: >16(F)/24(M) g/day, % | 19.3 | 11.7 | 10.5 | 8.6 |
| Diet pattern: Unhealthy^a^, % | 35.8 | 34.3 | 31.6 | 32.5 |
| Diet pattern: Sweet^a^, % | 9.2 | 16.4 | 24.8 | 21.8 |
| Diet pattern: Mediterranean-like^a^, % | 18.8 | 17.5 | 13.0 | 11.5 |
| Diet pattern: Healthy^a^, % | 36.1 | 31.8 | 30.6 | 34.2 |

Values are means ± SDs unless noted otherwise. ^a^ Diet pattern as identified in cluster analysis (Brunner EJ, Mosdøl A, Witte DR, et al. Dietary patterns and 15-y risks of major coronary events, diabetes, and mortality. *Am J Clin Nutr* 2008; 87: 1414–21).

**Table S2. Mortality during follow-up.**

|  | **Frequency of tinned fruit consumption** | | | |
| --- | --- | --- | --- | --- |
|  | <1 per month | 1-3 per month | 1 per week | ≥2 per week |
| **EPIC-Norfolk, 1993-2012** |  |  |  |  |
| Participants | 11,655 | 6260 | 3152 | 1355 |
| Person-years | 185 869 | 98 169 | 49 191 | 20 658 |
| Mortality, per 1000 person-years | 11.5 | 14.5 | 16.5 | 18.9 |
| Mortality | 2134 | 1422 | 812 | 391 |
| Cardiovascular | 617 | 461 | 277 | 135 |
| Cancer | 837 | 518 | 289 | 111 |
| Non-cardiovascular, non-cancer | 680 | 443 | 246 | 145 |
| **EPIC-Oxford, 1993-2012** |  |  |  |  |
| Participants | 34,795 | 11,594 | 4195 | 2041 |
| Person-years | 542 155 | 180 414 | 65 561 | 31 515 |
| Mortality, per 1000 person-years | 3.7 | 4.8 | 5.2 | 6.6 |
| Mortality | 1982 | 869 | 341 | 207 |
| Cardiovascular | 512 | 263 | 115 | 66 |
| Cancer | 893 | 356 | 116 | 64 |
| Non-cardiovascular, non-cancer | 577 | 250 | 110 | 77 |
| **Whitehall II, 1991-2012** |  |  |  |  |
| Participants | 4277 | 2015 | 800 | 348 |
| Person-years | 84 015 | 39 595 | 15 625 | 6 835 |
| Mortality, per 1000 person-years | 4.7 | 4.8 | 4.9 | 5.1 |
| Mortality | 397 | 189 | 77 | 35 |
| Cardiovascular | 102 | 51 | 26 | 10 |
| Cancer | 202 | 83 | 35 | 14 |
| Non-cardiovascular, non-cancer | 88 | 55 | 16 | 11 |

All values are numbers.

**Table S3. Sensitivity and subgroup analyses: Hazard ratios (95% CIs) for all cause mortality by tinned fruit consumption.**

| **Analysis by cohort** | **Frequency of tinned fruit consumption** | | | |  |
| --- | --- | --- | --- | --- | --- |
|  | <1/month | 1-3/month | 1/week | ≥2/week |  |
| **EPIC-Norfolk, 1993-2012** |  |  |  |  |  |
| Multivariable adjusted hazard ratio (N=21,978) | 1.00 | 1.02 (0.95, 1.09) | 1.13 (1.04, 1.23) | 1.16 (1.04, 1.30) |  |
| Excluding unusual BMIs (<18.5 or >40 kg/m^2^) (N=21,734) | 1.00 | 1.03 (0.96, 1.10) | 1.13 (1.04, 1.23) | 1.16 (1.03, 1.30) |  |
| Excluding diabetes, hypertensive drug use or lipid drug use (N=17,991) | 1.00 | 1.06 (0.98, 1.16) | 1.09 (0.99, 1.21) | 1.16 (1.01, 1.33) |  |
| Excluding deaths in first 2 years of follow-up (N=21 ,804) | 1.00 | 1.02 (0.95, 1.10) | 1.13 (1.04, 1.23) | 1.14 (1.02, 1.28) |  |
| Men (N=9901) | 1.00 | 0.99 (0.90, 1.09) | 1.11 (0.99, 1.25) | 1.19 (1.03, 1.38) |  |
| Women (N=12,077) | 1.00 | 1.05 (0.95, 1.17) | 1.15 (1.00, 1.30) | 1.11 (0.94, 1.32) |  |
| Non-obese: BMI <30 kg/m^2^ (N=18,732) | 1.00 | 1.03 (0.95, 1.11) | 1.12 (1.02, 1.23) | 1.15 (1.02, 1.30) |  |
| Obese: BMI ≥30 kg/m^2^ (N=3246) | 1.00 | 0.99 (0.84, 1.17) | 1.19 (0.98, 1.45) | 1.23 (0.94, 1.60) |  |
| Age <60 y (N=12,210) | 1.00 | 1.03 (0.88, 1.21) | 1.22 (1.00, 1.48) | 0.83 (0.59, 1.17) |  |
| Age ≥60 y (N=9768) | 1.00 | 1.02 (0.94, 1.10) | 1.11 (1.01, 1.22) | 1.22 (1.08, 1.37) |  |
| Updating FFQ data from up to 2 time points (N=21,977) | 1.00 | 1.04 (0.97, 1.11) | 1.12 (1.03, 1.22) | 1.13 (1.01, 1.25) |  |
| **EPIC-Oxford, 1993-2012** |  |  |  |  |  |
| Multivariable adjusted hazard ratio (N=52,625) | 1.00 | 1.11 (1.03, 1.21) | 1.07 (0.95, 1.21) | 1.09 (0.95, 1.27) |  |
| Excluding unusual BMIs (<18.5 or ≥40 kg/m^2^) (N=49,106) | 1.00 | 1.12 (1.03, 1.22) | 1.07 (0.94, 1.21) | 1.11 (0.96, 1.30) |  |
| Excluding diabetes and long-term medical treatment (N=39,958) | 1.00 | 1.10 (0.99, 1.22) | 1.07 (0.91, 1.25) | 1.09 (0.88, 1.34) |  |
| Excluding deaths in first 2 years of follow-up (N=52,355) | 1.00 | 1.11 (1.02, 1.21) | 1.08 (0.96, 1.22) | 1.09 (0.94, 1.27) |  |
| Men (N=11,966) | 1.00 | 1.09 (0.95, 1.26) | 1.11 (0.90, 1.35) | 0.89 (0.69, 1.13) |  |
| Women (N=40,659) | 1.00 | 1.11 (1.01, 1.23) | 1.06 (0.92, 1.23) | 1.24 (1.03, 1.49) |  |
| Non-obese: BMI <30 kg/m^2^ (N=47,677) | 1.00 | 1.11 (1.02, 1.21) | 1.04 (0.91, 1.18) | 1.10 (0.94, 1.29) |  |
| Obese: BMI ≥30 kg/m^2^ (N=3185) | 1.00 | 1.16 (0.88, 1.53) | 1.27 (0.88, 1.82) | 1.26 (0.78, 2.05) |  |
| Age <60 y (N=44,711) | 1.00 | 1.06 (0.93, 1.21) | 0.99 (0.81, 1.21) | 1.00 (0.76, 1.32) |  |
| Age ≥60 y (N=7914) | 1.00 | 1.14 (1.03, 1.27) | 1.12 (0.97, 1.30) | 1.15 (0.96, 1.36) |  |
| **Whitehall II, 1991-2012** |  |  |  |  |  |
| Multivariable adjusted hazard ratio (N=7440) | 1.00 | 1.00 (0.83, 1.19) | 0.98 (0.76, 1.27) | 1.04 (0.73, 1.48) |  |
| Excluding unusual BMIs (<18.5 or >40 kg/m^2^) (N=7326) | 1.00 | 1.00 (0.84, 1.20) | 0.96 (0.74, 1.24) | 1.06 (0.74, 1.51) |  |
| Excluding diabetes, hypertensive drug use or lipid drug use (N=6890) | 1.00 | 0.94 (0.78, 1.14) | 0.93 (0.71, 1.23) | 0.97 (0.66, 1.42) |  |
| Excluding deaths in first 2 years of follow-up (N=7419) | 1.00 | 1.01 (0.84, 1.21) | 0.94 (0.72, 1.22) | 1.09 (0.76, 1.55) |  |
| Men (N=5199) | 1.00 | 1.03 (0.83, 1.27) | 0.93 (0.69, 1.26) | 1.00 (0.67, 1.50) |  |
| Women (N=2241) | 1.00 | 0.88 (0.62, 1.26) | 1.15 (0.72, 1.85) | 1.15 (0.53, 2.50) |  |
| Non-obese: BMI <30 kg/m^2^ (N=6754) | 1.00 | 1.02 (0.84, 1.24) | 1.05 (0.80, 1.38) | 1.01 (0.68, 1.50) |  |
| Obese: BMI ≥30 kg/m^2^ (N=686) | 1.00 | 1.04 (0.64, 1.69) | 0.76 (0.37, 1.55) | 1.39 (0.64, 3.03) |  |
| Age <60 y (N=7100) | 1.00 | 1.08 (0.89, 1.30) | 1.03 (0.78, 1.34) | 1.05 (0.71, 1.55) |  |
| Age ≥60 y (N=340) | 1.00 | 0.72 (0.40, 1.30) | 1.04 (0.51, 2.11) | 1.10 (0.47, 2.58) |  |
| Using FFQ data from up to 2 time points (N=7440) | 1.00 | 1.05 (0.88, 1.26) | 1.07 (0.83, 1.38) | 1.24 (0.90, 1.72) |  |
| Using FFQ data from up to 3 time points (N=7440) | 1.00 | 1.06 (0.88, 1.27) | 1.13 (0.88, 1.47) | 1.19 (0.86, 1.65) |  |
| **Pooled results** |  |  |  |  |  |
| Multivariable adjusted hazard ratio (N=82,043) | 1.00 | 1.05 (0.99, 1.12) | 1.10 (1.03, 1.18) | 1.13 (1.04, 1.23) |  |
| Excluding unusual BMIs (<18.5 or ≥40 kg/m^2^) (N=78,166) | 1.00 | 1.06 (0.99, 1.13) | 1.10 (1.03, 1.18) | 1.14 (1.04, 1.24) |  |
| Excluding people with diabetes and people with hypertensive drug use or lipid drug use/on long-term medical treatment (N=68,439) | 1.00 | 1.06 (1.00, 1.13) | 1.07 (0.99, 1.17) | 1.12 (1.00, 1.25) |  |
| Excluding deaths in first 2 years of follow-up (N=81,578) | 1.00 | 1.06 (1.00, 1.12) | 1.10 (1.03, 1.18) | 1.12 (1.03, 1.23) |  |
| Men (N=27,066) | 1.00 | 1.02 (0.95, 1.10) | 1.09 (1.00, 1.20) | 1.05 (0.85, 1.29) |  |
| Women (N=54,977) | 1.00 | 1.07 (1.00, 1.15) | 1.11 (1.01, 1.22) | 1.17 (1.03, 1.32) |  |
| Non-obese: BMI <30 kg/m^2^ (N=73,163) | 1.00 | 1.06 (1.00, 1.12) | 1.09 (1.01, 1.17) | 1.12 (1.02, 1.24) |  |
| Obese: BMI ≥30 kg/m^2^ (N=7117) | 1.00 | 1.04 (0.90, 1.19) | 1.18 (0.99, 1.39) | 1.25 (1.00, 1.56) |  |
| Age <60 y (N=64,021) | 1.00 | 1.06 (0.96, 1.15) | 1.09 (0.95, 1.24) | 0.96 (0.79, 1.15) |  |
| Age ≥60 y (N=18,022) | 1.00 | 1.06 (0.94, 1.19) | 1.11 (1.03, 1.20) | 1.19 (1.08, 1.32) |  |

Hazard ratios are adjusted for the same covariates as in Table 2.

**Table S4. Fruit consumption (g/day) at baseline.**

|  | **Frequency of tinned fruit consumption** | | | |
| --- | --- | --- | --- | --- |
|  | <1 per month | 1-3 per month | 1 per week | ≥2 per week |
| **EPIC-Norfolk, 1993-1997** |  |  |  |  |
| Participants, N | 11,655 | 6260 | 3152 | 1355 |
| Total fruit consumption | 241 ± 191 | 237 ± 175 | 252 ± 186 | 311 ± 202 |
| Total non-tinned fruit consumption | 241 ± 191 | 228 ± 175 | 235 ± 186 | 248 ± 195 |
| Apples | 73 ± 78 | 68 ± 71 | 73 ± 73 | 74 ± 79 |
| Pears | 27 ± 45 | 26 ± 41 | 27 ± 43 | 30 ± 48 |
| Oranges, satsumas, mandarins | 43 ± 61 | 39 ± 55 | 41 ± 54 | 41 ± 58 |
| Grapefruit | 7.4 ± 20.3 | 7.1 ± 17.4 | 7.3 ± 18.6 | 8.9 ± 23.9 |
| Bananas | 47 ± 51 | 44 ± 47 | 45 ± 49 | 47 ± 53 |
| Grapes | 9.4 ± 24.4 | 8.8 ± 22.8 | 8.6 ± 21.4 | 8.5 ± 19.9 |
| Melon | 12 ± 30 | 12 ± 26 | 11 ± 24 | 12 ± 29 |
| Peaches, plums, apricots | 7.5 ± 16.8 | 7.4 ± 16.0 | 7.0 ± 15.9 | 7.7 ± 14.1 |
| Strawberries, raspberries, kiwi fruit | 12.1 ± 23.3 | 12.8 ± 23.4 | 13.1 ± 25.9 | 13.4 ± 22.5 |
| Dried fruit, raisins, prunes | 3.0 ± 8.2 | 3.1 ± 7.6 | 3.3 ± 8.2 | 4.2 ± 10.3 |
| **EPIC-Oxford, 1993-2001** |  |  |  |  |
| Participants, N | 34,795 | 11,594 | 4195 | 2041 |
| Total fruit consumption | 280 ± 227 | 283 ± 209 | 299 ± 212 | 386 ± 246 |
| Total non-tinned fruit consumption | 279 ± 227 | 275 ± 209 | 282 ± 212 | 318 ± 232 |
| Apples | 76 ± 82 | 73 ± 76 | 76 ± 79 | 80 ± 81 |
| Pears | 28 ± 48 | 28 ± 45 | 29 ± 45 | 37 ± 60 |
| Oranges, satsumas, mandarins | 55 ± 75 | 52 ± 68 | 53 ± 69 | 54 ± 70 |
| Grapefruit | 7.8 ± 18.9 | 7.9 ± 16.9 | 8.1 ± 15.8 | 11.0 ± 21.6 |
| Bananas | 56 ± 58 | 55 ± 54 | 57 ± 53 | 63 ± 58 |
| Grapes | 11.0 ± 23.3 | 11.3 ± 23.5 | 11.6 ± 23.8 | 13.0 ± 24.8 |
| Melon | 19 ± 42 | 21 ± 37 | 21 ± 41 | 27 ± 45 |
| Peaches, plums, apricots | 8.5 ± 16.4 | 8.7 ± 16.0 | 8.8 ± 15.8 | 10.6 ± 17.5 |
| Strawberries, raspberries, kiwi fruit | 11.8 ± 21.2 | 12.6 ± 21.2 | 12.6 ± 19.4 | 15.9 ± 25.1 |
| Dried fruit, raisins, prunes | 5.7 ± 11.7 | 5.9 ± 11.4 | 6.5 ± 11.3 | 8.2 ± 13.7 |
| **Whitehall II, 1991-1993** |  |  |  |  |
| Participants, N | 4277 | 2015 | 800 | 348 |
| Total fruit consumption | 199 ± 161 | 203 ± 145 | 213 ± 137 | 270 ± 154 |
| Total non-tinned fruit consumption | 199 ± 161 | 195 ± 145 | 195 ± 137 | 208 ± 147 |
| Apples | 69 ± 77 | 66 ± 67 | 66 ± 63 | 69 ± 68 |
| Pears | 17 ± 32 | 16 ± 29 | 17 ± 29 | 16 ± 28 |
| Oranges, satsumas, mandarins | 42 ± 59 | 40 ± 53 | 37 ± 42 | 41 ± 48 |
| Grapefruit | 6.0 ± 15.1 | 6.0 ± 13.8 | 6.4 ± 13.8 | 7.2 ± 13.8 |
| Bananas | 32 ± 40 | 34 ± 38 | 32 ± 36 | 37 ± 42 |
| Grapes | 6.8 ± 15.8 | 6.5 ± 13.6 | 6.7 ± 14.4 | 6.9 ± 12.4 |
| Melon | 10 ± 25 | 11 ± 18 | 12 ± 19 | 13 ± 26 |
| Peaches, plums, apricots | 5.3 ± 10.9 | 5.2 ± 10.2 | 5.1 ± 10.2 | 5.1 ± 9.8 |
| Strawberries, raspberries | 7.5 ± 12.7 | 8.0 ± 11.2 | 8.9 ± 14.0 | 8.7 ± 9.9 |
| Dried fruit, raisins, prunes | 2.5 ± 7.0 | 2.8 ± 6.2 | 3.5 ± 7.5 | 3.6 ± 6.3 |

Values are means ± SDs unless noted otherwise.

One serving of fruit was defined as 120 g for all fruits except grapefruit (80 g), bananas (100 g), grapes (50 g), melon (180 g), peaches (30 g), strawberries (40 g) and dried fruit (25 g).

EPIC, European Prospective Investigation into Cancer and Nutrition.
