# Supplementary material for: Tinned Fruit Consumption and Mortality in Three Prospective Cohorts
Source: PLoS One. 2015 Feb 25;10(2):e0117796. doi: 10.1371/journal.pone.0117796 (PMC4340615; doi:10.1371/journal.pone.0117796)
Supplement: S1 Table — (DOCX) [file pone.0117796.s002.docx]

**Table S1. Participant characteristics at baseline by cohort.**

| **Characteristic** | **Frequency of tinned fruit consumption** | | | |
| --- | --- | --- | --- | --- |
|  | <1 per month | 1-3 per month | 1 per week | ≥2 per week |
| **EPIC-Norfolk, 1993-1997** |  |  |  |  |
| Participants, N | 11,655 | 6259 | 3152 | 1355 |
| Male, % | 42.2 | 46.6 | 49.2 | 50.1 |
| Age (y) | 57.2 ± 9.1 | 58.9 ± 9.3 | 59.5 ± 9.2 | 60.9 ± 9.2 |
| Ethnicity, white, % | 99.7 | 99.6 | 99.7 | 99.4 |
| Body mass index (kg/m^2^) | 26.1 ± 3.9 | 26.4 ± 3.8 | 26.6 ± 3.9 | 26.6 ± 3.9 |
| Physically inactive, % | 27.6 | 29.9 | 30.7 | 36.2 |
| Current smoker, % | 12.5 | 11.3 | 10.9 | 9.0 |
| Education, none or primary, % | 31.4 | 38.3 | 44.6 | 46.9 |
| Prior diabetes mellitus, % | 1.8 | 2.0 | 2.3 | 3.4 |
| Antihypertensive drug use, % | 14.8 | 17.7 | 18.8 | 19.9 |
| Lipid lowering drug use, % | 1.0 | 1.0 | 1.3 | 1.5 |
| Parental history of heart attack, % | 36.3 | 36.2 | 35.5 | 35.5 |
| Parental history of cancer, % | 33.0 | 32.5 | 32.4 | 32.6 |
| Total energy intake (kJ/day) | 8110 ± 2331 | 8959 ± 2464 | 9417 ± 2683 | 9824 ± 2810 |
| Alcohol intake (g/day) | 8.3 ± 10.2 | 6.4 ± 8.8 | 5.4 ± 8.1 | 5.2 ± 7.6 |
| Plasma vitamin C (µmol/L) | 55 ± 20 | 52 ± 20 | 51 ± 20 | 52 ± 20 |
| **EPIC-Oxford, 1993-2001** |  |  |  |  |
| Participants, N | 34,795 | 11,594 | 4195 | 2041 |
| Male, % | 22.3 | 23.5 | 23.7 | 24.3 |
| Age (y) | 43.7 ± 13.4 | 44.9 ± 14.3 | 45.7 ± 14.6 | 46.4 ± 15.4 |
| Ethnicity, white, % | 97.8 | 98.2 | 98.4 | 98.6 |
| Body mass index (kg/m^2^) | 23.5 ± 3.7 | 23.9 ± 3.9 | 24.2 ± 4.0 | 24.2 ± 4.1 |
| Physically inactive, % | 23.3 | 24.1 | 25.8 | 27.1 |
| Current smoker, % | 12.2 | 9.9 | 9.9 | 9.7 |
| Education, O level or less, % | 11.6 | 15.3 | 20.5 | 20.7 |
| Prior diabetes mellitus, % | 1.2 | 1.3 | 2.0 | 2.6 |
| Receiving long-term medical treatment, % | 23.6 | 26.5 | 29.9 | 33.4 |
| Parental history of heart attack or cancer, % | 52.2 | 53.5 | 53.2 | 52.1 |
| Total energy intake (kJ/day) | 7778 ± 2158 | 8558 ± 2265 | 8857 ± 2336 | 9019 ± 2410 |
| Alcohol intake (g/day) | 10.5 ± 13.2 | 8.3 ± 11.5 | 7.3 ± 10.6 | 6.5 ± 10.4 |
| **Whitehall II, 1991-1993** |  |  |  |  |
| Participants, N | 4277 | 2015 | 800 | 348 |
| Male, % | 66.1 | 73.6 | 76.5 | 79.3 |
| Age (y) | 49.2 ± 6.0 | 49.7 ± 6.1 | 50.0 ± 6.0 | 49.6 ± 6.3 |
| Ethnicity, white, % | 90.6 | 91.3 | 95.0 | 96.0 |
| Body mass index (kg/m^2^) | 25.3 ± 3.8 | 25.2 ± 3.5 | 25.4 ± 3.6 | 25.1 ± 3.7 |
| Physically inactive, % | 18.5 | 14.3 | 15.1 | 16.7 |
| Current smoker, % | 15.0 | 12.2 | 12.4 | 11.8 |
| Education, none or primary, % | 35.6 | 39.6 | 41.2 | 43.4 |
| Low employment grade, % | 15.0 | 15.9 | 15.5 | 18.1 |
| Prior diabetes mellitus, % | 0.7 | 0.9 | 0.6 | 0.6 |
| Antihypertensive drug use, % | 6.2 | 5.8 | 7.8 | 6.3 |
| Lipid lowering drug use, % | 0.6 | 0.7 | 1.0 | 1.2 |
| Total energy intake (kJ/day) | 8152 ± 2314 | 9152 ± 2401 | 9540 ± 2434 | 9983 ± 2315 |
| Reported energy intake / estimated energy expenditure | 0.74 ± 0.22 | 0.82 ± 0.23 | 0.85 ± 0.25 | 0.89 ± 0.24 |
| Alcohol intake (g/day) | 13.0 ± 15.1 | 10.7 ± 14.2 | 9.3 ± 12.6 | 8.6 ± 11.5 |
| Alcohol intake: None, % | 16.4 | 19.8 | 22.9 | 24.7 |
| Alcohol intake: 1-8 g/day, % | 35.1 | 38.9 | 42.3 | 42.2 |
| Alcohol intake: 9-16(F)/24(M) g/day, % | 29.3 | 29.6 | 24.4 | 24.4 |
| Alcohol intake: >16(F)/24(M) g/day, % | 19.3 | 11.7 | 10.5 | 8.6 |
| Diet pattern: Unhealthy^a^, % | 35.8 | 34.3 | 31.6 | 32.5 |
| Diet pattern: Sweet^a^, % | 9.2 | 16.4 | 24.8 | 21.8 |
| Diet pattern: Mediterranean-like^a^, % | 18.8 | 17.5 | 13.0 | 11.5 |
| Diet pattern: Healthy^a^, % | 36.1 | 31.8 | 30.6 | 34.2 |

Values are means ± SDs unless noted otherwise. ^a^ Diet pattern as identified in cluster analysis (Brunner EJ, Mosdøl A, Witte DR, et al. Dietary patterns and 15-y risks of major coronary events, diabetes, and mortality. *Am J Clin Nutr* 2008; 87: 1414–21).
