# Supplementary material for: Tinned Fruit Consumption and Mortality in Three Prospective Cohorts
Source: PLoS One. 2015 Feb 25;10(2):e0117796. doi: 10.1371/journal.pone.0117796 (PMC4340615; doi:10.1371/journal.pone.0117796)
Supplement: S2 Table — (DOCX) [file pone.0117796.s003.docx]

**Table S2. Mortality during follow-up.**

|  | **Frequency of tinned fruit consumption** | | | |
| --- | --- | --- | --- | --- |
|  | <1 per month | 1-3 per month | 1 per week | ≥2 per week |
| **EPIC-Norfolk, 1993-2012** |  |  |  |  |
| Participants | 11,655 | 6260 | 3152 | 1355 |
| Person-years | 185 869 | 98 169 | 49 191 | 20 658 |
| Mortality, per 1000 person-years | 11.5 | 14.5 | 16.5 | 18.9 |
| Mortality | 2134 | 1422 | 812 | 391 |
| Cardiovascular | 617 | 461 | 277 | 135 |
| Cancer | 837 | 518 | 289 | 111 |
| Non-cardiovascular, non-cancer | 680 | 443 | 246 | 145 |
| **EPIC-Oxford, 1993-2012** |  |  |  |  |
| Participants | 34,795 | 11,594 | 4195 | 2041 |
| Person-years | 542 155 | 180 414 | 65 561 | 31 515 |
| Mortality, per 1000 person-years | 3.7 | 4.8 | 5.2 | 6.6 |
| Mortality | 1982 | 869 | 341 | 207 |
| Cardiovascular | 512 | 263 | 115 | 66 |
| Cancer | 893 | 356 | 116 | 64 |
| Non-cardiovascular, non-cancer | 577 | 250 | 110 | 77 |
| **Whitehall II, 1991-2012** |  |  |  |  |
| Participants | 4277 | 2015 | 800 | 348 |
| Person-years | 84 015 | 39 595 | 15 625 | 6 835 |
| Mortality, per 1000 person-years | 4.7 | 4.8 | 4.9 | 5.1 |
| Mortality | 397 | 189 | 77 | 35 |
| Cardiovascular | 102 | 51 | 26 | 10 |
| Cancer | 202 | 83 | 35 | 14 |
| Non-cardiovascular, non-cancer | 88 | 55 | 16 | 11 |

All values are numbers.
