# Supplementary material for: Tinned Fruit Consumption and Mortality in Three Prospective Cohorts
Source: PLoS One. 2015 Feb 25;10(2):e0117796. doi: 10.1371/journal.pone.0117796 (PMC4340615; doi:10.1371/journal.pone.0117796)
Supplement: S3 Table — (DOCX) [file pone.0117796.s004.docx]

**Table S3. Sensitivity and subgroup analyses: Hazard ratios (95% CIs) for all cause mortality by tinned fruit consumption.**

| **Analysis by cohort** | **Frequency of tinned fruit consumption** | | | |  |
| --- | --- | --- | --- | --- | --- |
|  | <1/month | 1-3/month | 1/week | ≥2/week |  |
| **EPIC-Norfolk, 1993-2012** |  |  |  |  |  |
| Multivariable adjusted hazard ratio (N=21,978) | 1.00 | 1.02 (0.95, 1.09) | 1.13 (1.04, 1.23) | 1.16 (1.04, 1.30) |  |
| Excluding unusual BMIs (<18.5 or >40 kg/m^2^) (N=21,734) | 1.00 | 1.03 (0.96, 1.10) | 1.13 (1.04, 1.23) | 1.16 (1.03, 1.30) |  |
| Excluding diabetes, hypertensive drug use or lipid drug use (N=17,991) | 1.00 | 1.06 (0.98, 1.16) | 1.09 (0.99, 1.21) | 1.16 (1.01, 1.33) |  |
| Excluding deaths in first 2 years of follow-up (N=21 ,804) | 1.00 | 1.02 (0.95, 1.10) | 1.13 (1.04, 1.23) | 1.14 (1.02, 1.28) |  |
| Men (N=9901) | 1.00 | 0.99 (0.90, 1.09) | 1.11 (0.99, 1.25) | 1.19 (1.03, 1.38) |  |
| Women (N=12,077) | 1.00 | 1.05 (0.95, 1.17) | 1.15 (1.00, 1.30) | 1.11 (0.94, 1.32) |  |
| Non-obese: BMI <30 kg/m^2^ (N=18,732) | 1.00 | 1.03 (0.95, 1.11) | 1.12 (1.02, 1.23) | 1.15 (1.02, 1.30) |  |
| Obese: BMI ≥30 kg/m^2^ (N=3246) | 1.00 | 0.99 (0.84, 1.17) | 1.19 (0.98, 1.45) | 1.23 (0.94, 1.60) |  |
| Age <60 y (N=12,210) | 1.00 | 1.03 (0.88, 1.21) | 1.22 (1.00, 1.48) | 0.83 (0.59, 1.17) |  |
| Age ≥60 y (N=9768) | 1.00 | 1.02 (0.94, 1.10) | 1.11 (1.01, 1.22) | 1.22 (1.08, 1.37) |  |
| Updating FFQ data from up to 2 time points (N=21,977) | 1.00 | 1.04 (0.97, 1.11) | 1.12 (1.03, 1.22) | 1.13 (1.01, 1.25) |  |
| **EPIC-Oxford, 1993-2012** |  |  |  |  |  |
| Multivariable adjusted hazard ratio (N=52,625) | 1.00 | 1.11 (1.03, 1.21) | 1.07 (0.95, 1.21) | 1.09 (0.95, 1.27) |  |
| Excluding unusual BMIs (<18.5 or ≥40 kg/m^2^) (N=49,106) | 1.00 | 1.12 (1.03, 1.22) | 1.07 (0.94, 1.21) | 1.11 (0.96, 1.30) |  |
| Excluding diabetes and long-term medical treatment (N=39,958) | 1.00 | 1.10 (0.99, 1.22) | 1.07 (0.91, 1.25) | 1.09 (0.88, 1.34) |  |
| Excluding deaths in first 2 years of follow-up (N=52,355) | 1.00 | 1.11 (1.02, 1.21) | 1.08 (0.96, 1.22) | 1.09 (0.94, 1.27) |  |
| Men (N=11,966) | 1.00 | 1.09 (0.95, 1.26) | 1.11 (0.90, 1.35) | 0.89 (0.69, 1.13) |  |
| Women (N=40,659) | 1.00 | 1.11 (1.01, 1.23) | 1.06 (0.92, 1.23) | 1.24 (1.03, 1.49) |  |
| Non-obese: BMI <30 kg/m^2^ (N=47,677) | 1.00 | 1.11 (1.02, 1.21) | 1.04 (0.91, 1.18) | 1.10 (0.94, 1.29) |  |
| Obese: BMI ≥30 kg/m^2^ (N=3185) | 1.00 | 1.16 (0.88, 1.53) | 1.27 (0.88, 1.82) | 1.26 (0.78, 2.05) |  |
| Age <60 y (N=44,711) | 1.00 | 1.06 (0.93, 1.21) | 0.99 (0.81, 1.21) | 1.00 (0.76, 1.32) |  |
| Age ≥60 y (N=7914) | 1.00 | 1.14 (1.03, 1.27) | 1.12 (0.97, 1.30) | 1.15 (0.96, 1.36) |  |
| **Whitehall II, 1991-2012** |  |  |  |  |  |
| Multivariable adjusted hazard ratio (N=7440) | 1.00 | 1.00 (0.83, 1.19) | 0.98 (0.76, 1.27) | 1.04 (0.73, 1.48) |  |
| Excluding unusual BMIs (<18.5 or >40 kg/m^2^) (N=7326) | 1.00 | 1.00 (0.84, 1.20) | 0.96 (0.74, 1.24) | 1.06 (0.74, 1.51) |  |
| Excluding diabetes, hypertensive drug use or lipid drug use (N=6890) | 1.00 | 0.94 (0.78, 1.14) | 0.93 (0.71, 1.23) | 0.97 (0.66, 1.42) |  |
| Excluding deaths in first 2 years of follow-up (N=7419) | 1.00 | 1.01 (0.84, 1.21) | 0.94 (0.72, 1.22) | 1.09 (0.76, 1.55) |  |
| Men (N=5199) | 1.00 | 1.03 (0.83, 1.27) | 0.93 (0.69, 1.26) | 1.00 (0.67, 1.50) |  |
| Women (N=2241) | 1.00 | 0.88 (0.62, 1.26) | 1.15 (0.72, 1.85) | 1.15 (0.53, 2.50) |  |
| Non-obese: BMI <30 kg/m^2^ (N=6754) | 1.00 | 1.02 (0.84, 1.24) | 1.05 (0.80, 1.38) | 1.01 (0.68, 1.50) |  |
| Obese: BMI ≥30 kg/m^2^ (N=686) | 1.00 | 1.04 (0.64, 1.69) | 0.76 (0.37, 1.55) | 1.39 (0.64, 3.03) |  |
| Age <60 y (N=7100) | 1.00 | 1.08 (0.89, 1.30) | 1.03 (0.78, 1.34) | 1.05 (0.71, 1.55) |  |
| Age ≥60 y (N=340) | 1.00 | 0.72 (0.40, 1.30) | 1.04 (0.51, 2.11) | 1.10 (0.47, 2.58) |  |
| Using FFQ data from up to 2 time points (N=7440) | 1.00 | 1.05 (0.88, 1.26) | 1.07 (0.83, 1.38) | 1.24 (0.90, 1.72) |  |
| Using FFQ data from up to 3 time points (N=7440) | 1.00 | 1.06 (0.88, 1.27) | 1.13 (0.88, 1.47) | 1.19 (0.86, 1.65) |  |
| **Pooled results** |  |  |  |  |  |
| Multivariable adjusted hazard ratio (N=82,043) | 1.00 | 1.05 (0.99, 1.12) | 1.10 (1.03, 1.18) | 1.13 (1.04, 1.23) |  |
| Excluding unusual BMIs (<18.5 or ≥40 kg/m^2^) (N=78,166) | 1.00 | 1.06 (0.99, 1.13) | 1.10 (1.03, 1.18) | 1.14 (1.04, 1.24) |  |
| Excluding people with diabetes and people with hypertensive drug use or lipid drug use/on long-term medical treatment (N=68,439) | 1.00 | 1.06 (1.00, 1.13) | 1.07 (0.99, 1.17) | 1.12 (1.00, 1.25) |  |
| Excluding deaths in first 2 years of follow-up (N=81,578) | 1.00 | 1.06 (1.00, 1.12) | 1.10 (1.03, 1.18) | 1.12 (1.03, 1.23) |  |
| Men (N=27,066) | 1.00 | 1.02 (0.95, 1.10) | 1.09 (1.00, 1.20) | 1.05 (0.85, 1.29) |  |
| Women (N=54,977) | 1.00 | 1.07 (1.00, 1.15) | 1.11 (1.01, 1.22) | 1.17 (1.03, 1.32) |  |
| Non-obese: BMI <30 kg/m^2^ (N=73,163) | 1.00 | 1.06 (1.00, 1.12) | 1.09 (1.01, 1.17) | 1.12 (1.02, 1.24) |  |
| Obese: BMI ≥30 kg/m^2^ (N=7117) | 1.00 | 1.04 (0.90, 1.19) | 1.18 (0.99, 1.39) | 1.25 (1.00, 1.56) |  |
| Age <60 y (N=64,021) | 1.00 | 1.06 (0.96, 1.15) | 1.09 (0.95, 1.24) | 0.96 (0.79, 1.15) |  |
| Age ≥60 y (N=18,022) | 1.00 | 1.06 (0.94, 1.19) | 1.11 (1.03, 1.20) | 1.19 (1.08, 1.32) |  |

Hazard ratios are adjusted for the same covariates as in Table 2.
