# Supplementary material for: Tinned Fruit Consumption and Mortality in Three Prospective Cohorts
Source: PLoS One. 2015 Feb 25;10(2):e0117796. doi: 10.1371/journal.pone.0117796 (PMC4340615; doi:10.1371/journal.pone.0117796)
Supplement: S4 Table — (DOCX) [file pone.0117796.s005.docx]

**Table S4. Fruit consumption at baseline, shown in grams per day.**

|  | **Frequency of tinned fruit consumption** | | | |
| --- | --- | --- | --- | --- |
|  | <1 per month | 1-3 per month | 1 per week | ≥2 per week |
| **EPIC-Norfolk, 1993-1997** |  |  |  |  |
| Participants, N | 11,655 | 6260 | 3152 | 1355 |
| Total fruit consumption | 241 ± 191 | 237 ± 175 | 252 ± 186 | 311 ± 202 |
| Total non-tinned fruit consumption | 241 ± 191 | 228 ± 175 | 235 ± 186 | 248 ± 195 |
| Apples | 73 ± 78 | 68 ± 71 | 73 ± 73 | 74 ± 79 |
| Pears | 27 ± 45 | 26 ± 41 | 27 ± 43 | 30 ± 48 |
| Oranges, satsumas, mandarins | 43 ± 61 | 39 ± 55 | 41 ± 54 | 41 ± 58 |
| Grapefruit | 7.4 ± 20.3 | 7.1 ± 17.4 | 7.3 ± 18.6 | 8.9 ± 23.9 |
| Bananas | 47 ± 51 | 44 ± 47 | 45 ± 49 | 47 ± 53 |
| Grapes | 9.4 ± 24.4 | 8.8 ± 22.8 | 8.6 ± 21.4 | 8.5 ± 19.9 |
| Melon | 12 ± 30 | 12 ± 26 | 11 ± 24 | 12 ± 29 |
| Peaches, plums, apricots | 7.5 ± 16.8 | 7.4 ± 16.0 | 7.0 ± 15.9 | 7.7 ± 14.1 |
| Strawberries, raspberries, kiwi fruit | 12.1 ± 23.3 | 12.8 ± 23.4 | 13.1 ± 25.9 | 13.4 ± 22.5 |
| Dried fruit, raisins, prunes | 3.0 ± 8.2 | 3.1 ± 7.6 | 3.3 ± 8.2 | 4.2 ± 10.3 |
| **EPIC-Oxford, 1993-2001** |  |  |  |  |
| Participants, N | 34,795 | 11,594 | 4195 | 2041 |
| Total fruit consumption | 280 ± 227 | 283 ± 209 | 299 ± 212 | 386 ± 246 |
| Total non-tinned fruit consumption | 279 ± 227 | 275 ± 209 | 282 ± 212 | 318 ± 232 |
| Apples | 76 ± 82 | 73 ± 76 | 76 ± 79 | 80 ± 81 |
| Pears | 28 ± 48 | 28 ± 45 | 29 ± 45 | 37 ± 60 |
| Oranges, satsumas, mandarins | 55 ± 75 | 52 ± 68 | 53 ± 69 | 54 ± 70 |
| Grapefruit | 7.8 ± 18.9 | 7.9 ± 16.9 | 8.1 ± 15.8 | 11.0 ± 21.6 |
| Bananas | 56 ± 58 | 55 ± 54 | 57 ± 53 | 63 ± 58 |
| Grapes | 11.0 ± 23.3 | 11.3 ± 23.5 | 11.6 ± 23.8 | 13.0 ± 24.8 |
| Melon | 19 ± 42 | 21 ± 37 | 21 ± 41 | 27 ± 45 |
| Peaches, plums, apricots | 8.5 ± 16.4 | 8.7 ± 16.0 | 8.8 ± 15.8 | 10.6 ± 17.5 |
| Strawberries, raspberries, kiwi fruit | 11.8 ± 21.2 | 12.6 ± 21.2 | 12.6 ± 19.4 | 15.9 ± 25.1 |
| Dried fruit, raisins, prunes | 5.7 ± 11.7 | 5.9 ± 11.4 | 6.5 ± 11.3 | 8.2 ± 13.7 |
| **Whitehall II, 1991-1993** |  |  |  |  |
| Participants, N | 4277 | 2015 | 800 | 348 |
| Total fruit consumption | 199 ± 161 | 203 ± 145 | 213 ± 137 | 270 ± 154 |
| Total non-tinned fruit consumption | 199 ± 161 | 195 ± 145 | 195 ± 137 | 208 ± 147 |
| Apples | 69 ± 77 | 66 ± 67 | 66 ± 63 | 69 ± 68 |
| Pears | 17 ± 32 | 16 ± 29 | 17 ± 29 | 16 ± 28 |
| Oranges, satsumas, mandarins | 42 ± 59 | 40 ± 53 | 37 ± 42 | 41 ± 48 |
| Grapefruit | 6.0 ± 15.1 | 6.0 ± 13.8 | 6.4 ± 13.8 | 7.2 ± 13.8 |
| Bananas | 32 ± 40 | 34 ± 38 | 32 ± 36 | 37 ± 42 |
| Grapes | 6.8 ± 15.8 | 6.5 ± 13.6 | 6.7 ± 14.4 | 6.9 ± 12.4 |
| Melon | 10 ± 25 | 11 ± 18 | 12 ± 19 | 13 ± 26 |
| Peaches, plums, apricots | 5.3 ± 10.9 | 5.2 ± 10.2 | 5.1 ± 10.2 | 5.1 ± 9.8 |
| Strawberries, raspberries | 7.5 ± 12.7 | 8.0 ± 11.2 | 8.9 ± 14.0 | 8.7 ± 9.9 |
| Dried fruit, raisins, prunes | 2.5 ± 7.0 | 2.8 ± 6.2 | 3.5 ± 7.5 | 3.6 ± 6.3 |

Values are means ± SDs unless noted otherwise.

One serving of fruit was defined as 120 g for all fruits except grapefruit (80 g), bananas (100 g), grapes (50 g), melon (180 g), peaches (30 g), strawberries (40 g) and dried fruit (25 g).

EPIC, European Prospective Investigation into Cancer and Nutrition.
